# Evolution and Development of Ventricular Septation in the Amniote Heart

**DOI:** 10.1371/journal.pone.0106569

**Published:** 2014-09-05

**Authors:** Robert E. Poelmann, Adriana C. Gittenberger-de Groot, Rebecca Vicente-Steijn, Lambertus J. Wisse, Margot M. Bartelings, Sonja Everts, Tamara Hoppenbrouwers, Boudewijn P. T. Kruithof, Bjarke Jensen, Paul W. de Bruin, Tatsuya Hirasawa, Shigeru Kuratani, Freek Vonk, Jeanne M. M. S. van de Put, Merijn A. de Bakker, Michael K. Richardson

**Affiliations:** 1 Department of Anatomy and Embryology, Leiden University Medical Center, Leiden, The Netherlands; 2 Department of Cardiology, Leiden University Medical Center, Leiden, The Netherlands; 3 Department of Molecular Cell Biology, Leiden University Medical Center, Leiden, The Netherlands; 4 Department of Anatomy, Embryology and Physiology, AMC Amsterdam, Amsterdam, The Netherlands; 5 Department of Bioscience-Zoophysiology, Aarhus University, Aarhus, Denmark; 6 Department of Radiology, Leiden University Medical Center, Leiden, The Netherlands; 7 Laboratory for Evolutionary Morphology, RIKEN Center for Developmental Biology, Kobe, Japan; 8 Naturalis Biodiversity Center, Darwinweg 2, Leiden, The Netherlands; 9 Institute of Biology Leiden (IBL), Leiden University, Sylvius Laboratory, Leiden, The Netherlands; New York Medical College, United States of America

## Abstract

During cardiogenesis the epicardium, covering the surface of the myocardial tube, has been ascribed several functions essential for normal heart development of vertebrates from lampreys to mammals. We investigated a novel function of the epicardium in ventricular development in species with partial and complete septation. These species include reptiles, birds and mammals. Adult turtles, lizards and snakes have a complex ventricle with three cava, partially separated by the horizontal and vertical septa. The crocodilians, birds and mammals with origins some 100 million years apart, however, have a left and right ventricle that are completely separated, being a clear example of convergent evolution. In specific embryonic stages these species show similarities in development, prompting us to investigate the mechanisms underlying epicardial involvement. The primitive ventricle of early embryos becomes septated by folding and fusion of the anterior ventricular wall, trapping epicardium in its core. This folding septum develops as the horizontal septum in reptiles and the anterior part of the interventricular septum in the other taxa. The mechanism of folding is confirmed using DiI tattoos of the ventricular surface. Trapping of epicardium-derived cells is studied by transplanting embryonic quail pro-epicardial organ into chicken hosts. The effect of decreased epicardium involvement is studied in knock-out mice, and pro-epicardium ablated chicken, resulting in diminished and even absent septum formation. Proper folding followed by diminished ventricular fusion may explain the deep interventricular cleft observed in elephants. The vertical septum, although indistinct in most reptiles except in crocodilians and pythonidsis apparently homologous to the inlet septum. Eventually the various septal components merge to form the completely septated heart. In our attempt to discover homologies between the various septum components we aim to elucidate the evolution and development of this part of the vertebrate heart as well as understand the etiology of septal defects in human congenital heart malformations.

## Introduction

**Figure 1:**
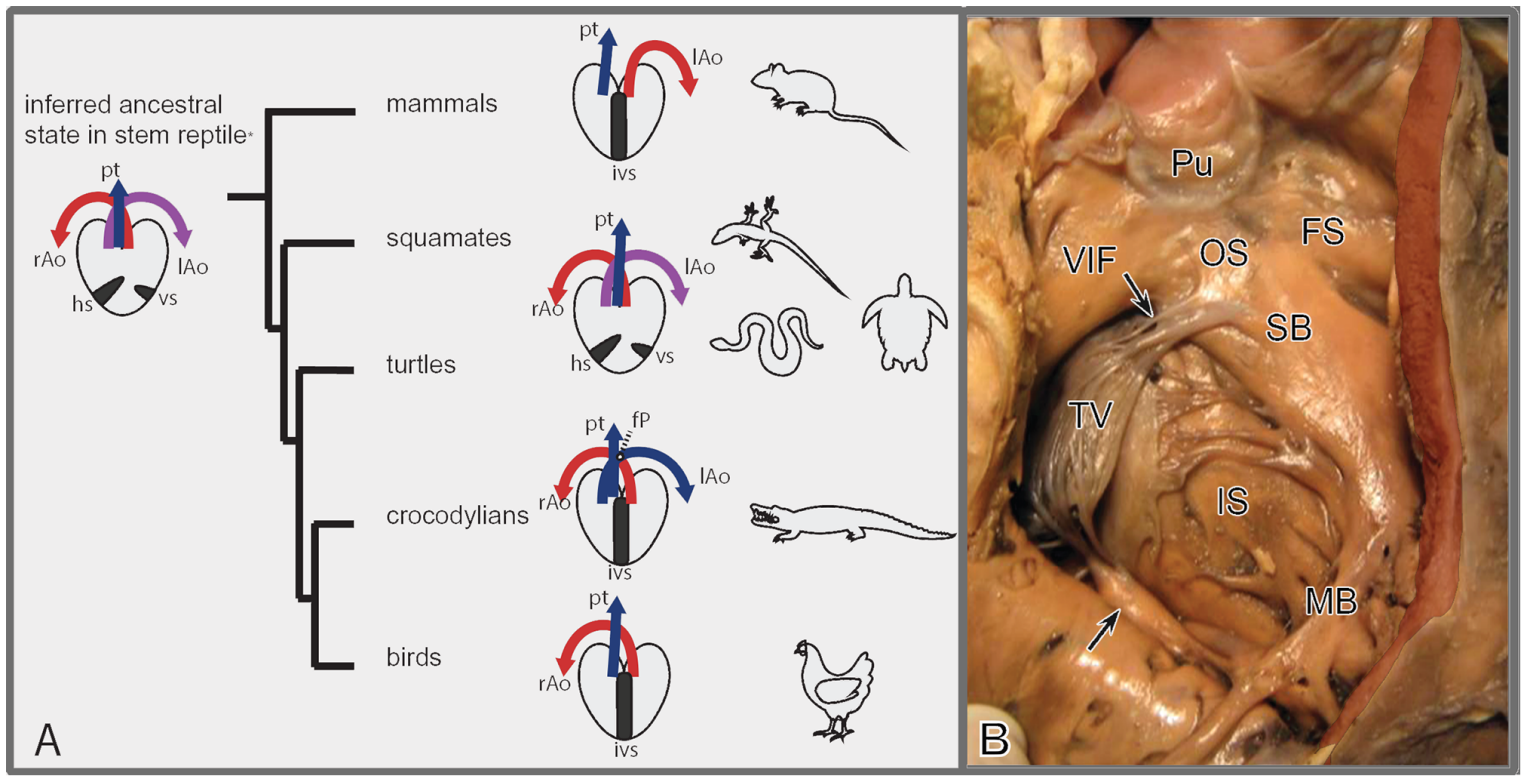
Evolution and septation of the heart. A. Evolution of hearts in higher vertebrates. Archosaurs (crocodilians, birds) and mammals independently evolved complete ventricular septation. Birds and mammals have lost either a left (lAo) or right (rAo) aorta. The horizontal (hs) and vertical septum (vs) are schematically indicated, together with the pulmonary trunk (Pt). The evolutionary tree is based on ref (2). B. Septum components in the human heart. Right face of the septum in a human heart after opening the right ventricle (RV), with inlet and folding components. Dissection line of the RV free wall in pink. Abbreviations: FS folding septum, IS inlet septum; MB moderator band; Pu pulmonary semilunar valve leaflets; SB septal band; TV anterior tricuspid valve leaflet with chordae tendineae (arrows) connected to SB and IS.; VIF ventriculo-infundibular fold. Fig. courtesy dr. L. Houyel.

During evolution the ventricle of the heart became divided into left and right chambers by a complete septum. Interestingly, ventricular septation evolved independently in mammals and in the archosaurs, comprising birds and crocodilians [1] [2] (**Fig. 1A**). It is therefore, a textbook case of convergent evolution. Not only is cardiac ventricular septation of great intrinsic interest to evolutionary biologists, it is also crucial for the understanding of many types of heart defects in humans [3]. Our studies show that part of the septum is critically dependent on interactions between myocardium and the epicardium including the epicardium-derived cells (EPDCs) [4], [5] for its development, and will develop abnormally if the epicardium is disturbed [6], [7].

Cardiac ventricular development in vertebrates, starting in an embryonic state with a primitive common ventricular tube, leading to separation into the left (LV) and right ventricle (RV), is a complex phenomenon. The mechanisms involve both the ventricular inflow and outflow compartment [8]–[10]. Complete cardiac septation is found in mammals as well as in crocodilians and birds. Other extant reptile groups including the squamates (lizards and snakes) and chelonians (turtles) show what is assumed to be a primitive pattern. They show partial septation by a horizontal and a vertical septum leading to a ventricle that is divided into 3 interconnected cavities. The cavities are located sinistro-dorsal (the cavum arteriosum and cavum venosum unified as cavum dorsale) and dextro-ventral (the cavum ventrale, more often named the cavum pulmonale). Controversy exists on the homology of the cardiac chambers and the septal components and how the primitive reptilian pattern was modified into the complete septum in mammals, birds and crocodiles. Reptilian cardiac development has been addressed [2], [11]–[13], with regard to the horizontal and vertical septum [14]. It is not possible to reconstruct the evolutionary history of ventricular septation as fossil records of embryonic soft tissues are non-existent.

Our strategy was to investigate the pattern and mechanisms of ventricular septation across the higher vertebrates using multiple lines of evidence. We focused on the functional role of the epicardium and EPDCs using various approaches: 1. immunohistochemistry of epicardium and EPDCs in embryos of different vertebrate species (lizard, snake, turtle, chicken, mouse, human), 2. quail-chicken chimeras by transplantation of early quail pro-epicardial organ (PEO) into the pericardial cavity of chicken to label subsets of epicardial and endothelial cells, 3. epicardium-deficient animal models such as the podoplanin knockout mouse, and epicardial ablation experiments in chicken embryos, 4. DiI-labelling experiments of the myocardial surface in chicken embryos to analyze outgrowth of the cardiac compartments. 5. expression of Tbx5 in embryos of several species, paying attention to the different septal components. *Tbx5* is reported to show gradients along the cardiac tube for various amniote embryos [14], and to be highly enriched in the left ventricle and the left face of the interventricular septum [15], 6.dissection of the extremely deep interventricular sulcus in adult elephant hearts [16].

To visualize the developmental anatomy of the different septal components we made animated 3D reconstructions of embryonic hearts from a range of species, including humans.

## Results

### Septum components in the completely septated heart

Very heterogeneous terminology is used for components of the ventricular septum and their respective boundaries and we adapted the following(**Table 1** **,** **Fig. 1B**). The *inlet septum* is the posterior (or dorsal in prone animals) component of the interventricular septum between the left and right atrioventricular junctions. The *folding septum* (a new term introduced in this paper) is the anterior (or ventral in prone animals) component. The septal band is a muscular profile on the RV septal surface situated between the inlet and folding septa. The outflow tract (OFT) or aorto-pulmonary septum depending highly on neural crest contribution, differs considerably among species. In the completely septated heart it is the last component that seals the interventricular communication. Development of the aorto-pulmonary component of the ventricular septum [8] has not been specifically studied here.

**Table 1:** Index for the terminology used.

*Level of septation*	Non-crocodilian reptiles	Avian, mouse, human
***Outflow tract*** 1 *at arterial, semilunar valve and intracardiac levels*	Aorto-pulmonary septum	Aorto-pulmonary septum/conotruncal septum/OFT septum
***Primary or bulboventricular fold^2^***	Primary or bulboventricular fold	Primary or bulboventricular fold
Ventricular levels		
*Anterior^3^*	Horizontal/folding septum	Anterior/primary/folding septum
*Apical^4^*	When present: trabeculations	Apical trabecular septum
*Posterior^5^*	When present: Vertical septum	Inlet septum
***Atrioventricular canal^6^***		Membranous atrio- and interventricular septum

The numbers refer to the ^superscripts^ in the Table.

1 . Combinations of aortic sac, truncus, conus and bulbus have been used to describe this segment. Conus and bulbus are usually myocardial, whereas aortic sac and truncus refer mostly to the vascular part. Septation from the vascular, semilunar valve and intracardiac levels is interchangeably referred to as either aorto-pulmonary septum or outflow tract (OFT) septum. The endocardial cushions in the proximal intracardiac part myocardialize through induction by neural crest cells forming the aorto-pulmonary or OFT septum. The distal part of the cushions is remodelled into semilunar valves that are separated by fibrous tissue between the orifices of the great arteries. In reptiles the aorto-pulmonary septum is branched and separates the two aortae and the pulmonary trunk. In mammals the distinction between proximal and distal endocardial cushions is inconspicuous.

2. Bulboventricular fold, synonymous with the primary fold, between outflow and inlet portion of the primitive ventricle.

3. Anterior (positional), primary (time-related) and folding (mechanistic, new in this paper) septum are synonymously used. The bulboventricular fold extends apically over the anterior surface of the heart and deepens to enclose epicardium and subepicardial tissue, thus forming an anteriorly located folding septum. The folding septum is considered to be homologous to the reptilian horizontal septum, which is also called the muscular ridge (see for further synonyms ref 13).

4. The apical trabecular septum develops from the coalescence of many trabeculations and does not show a clear demarcation with the folding septum or the inlet septum.

5. The inlet septum in early stages of eventually completely septated hearts and in some reptiles (presence is species-dependent) is a dense muscular structure on the posterior wall of the ventricle without an infolding mechanism. In the current study we have clearly shown that the anterior margin of the inlet septum with the folding septum is formed by the septal band or trabecula septomarginalis. In earlier literature the septal band has been described as the posterior margin of the primary (or folding) septum.

6. The superior and inferior atrioventricular endocardial cushions fuse in the midline. In the central part the cushions are remodelled into fibrous (membranous) tissue that becomes part of the fibrous heart skeleton. Part of this forms the membranous septum which is located between right atrium and outflow of the left ventricle (atrio-ventricular component) and the remainder between RV and LV (interventricular component).This tissue is obliquely embedded in both the atrial and ventricular septa and as such is sometimes referred to as atrioventricular septum.

### The presence of the epicardium in the various species

**Figure 2:**
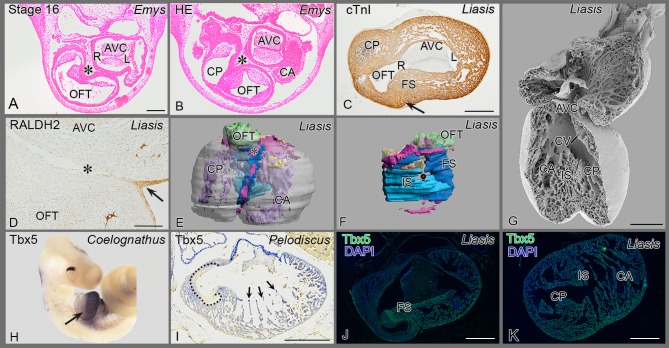
Reptile cardiac development. (**A, B**) The epicardial cushion (*) is located between OFT and AV cushions. (**C, D**) cardiac troponin I (cardiac muscle) and RALDH2 (epicardial cells) stainings show folding septum (arrow, asterisk). (**E**) 3D reconstruction in an anterior view, the epicardial patches are depicted in pink. See also Figure S1 1 for full animation. (**F**) right sided view of the septum, folding (FS) and inlet (IS) septum are depicted in shades of blue. For further colors see legend to Fig. 5E. (**G**) Scanning electron microscopy of anterior inner face, note communication between the three cava. The folding (syn. horizontal) septum is out of view. (**H**) A sharp decline of Tbx5 mRNA expression (arrow) between cavum dorsale and OFT. (**I**) Sharp boundary at muscular OFT (inside of dotted line) and wall of cavum pulmonale (outside dotted line), but the tip of trabeculations in the cavum dorsale stain strongly (arrows). (**J**) Section downstream of Fig C, showing sharp decline of Tbx5 protein expression at folding septum. (**K**) Section more to the apex of J, showing uniform immunostaining for Tbx5. Abbreviations as in Fig 1, others: AVC: AV cushions; ca, cp, cv: cavum arteriosum, pulmonale and venosum; L left AV orifice; OFT outflow tract cushions; R right AV orifice; →: infolding; * epicardium and EPDCs; • position of cavum venosum in 3D reconstruction of Fig F.

To infer primitive conditions in amniotes we examined embryos of the copperhead rat snake (*Coelognathus radiates*), Macklot's python (*Liasis mackloti*) (see also **Figure S1**), bearded dragon *(Pogona vitticeps)*, European pond turtle *(Emys orbicularis)*, and Chinese soft-shell turtle (*Pelodiscus sinensis*). The presence of epicardium, covering the outer face of the myocardial hart tube is confirmed. A pronounced subepicardium in the inner curvature of the looping heart tube at the site of the bulboventricular fold is noted (**Fig. 2A-D** **)** while in e.g. the turtle *Emys* **(** **Fig. 2A, B** **),** harboring an extensive loop of the OFT [17], the subepicardium, here referred to as epicardial cushion, is almost as elaborate as the flanking endocardial atrioventricular (AV) and OFT cushions.

**Figure 3:**
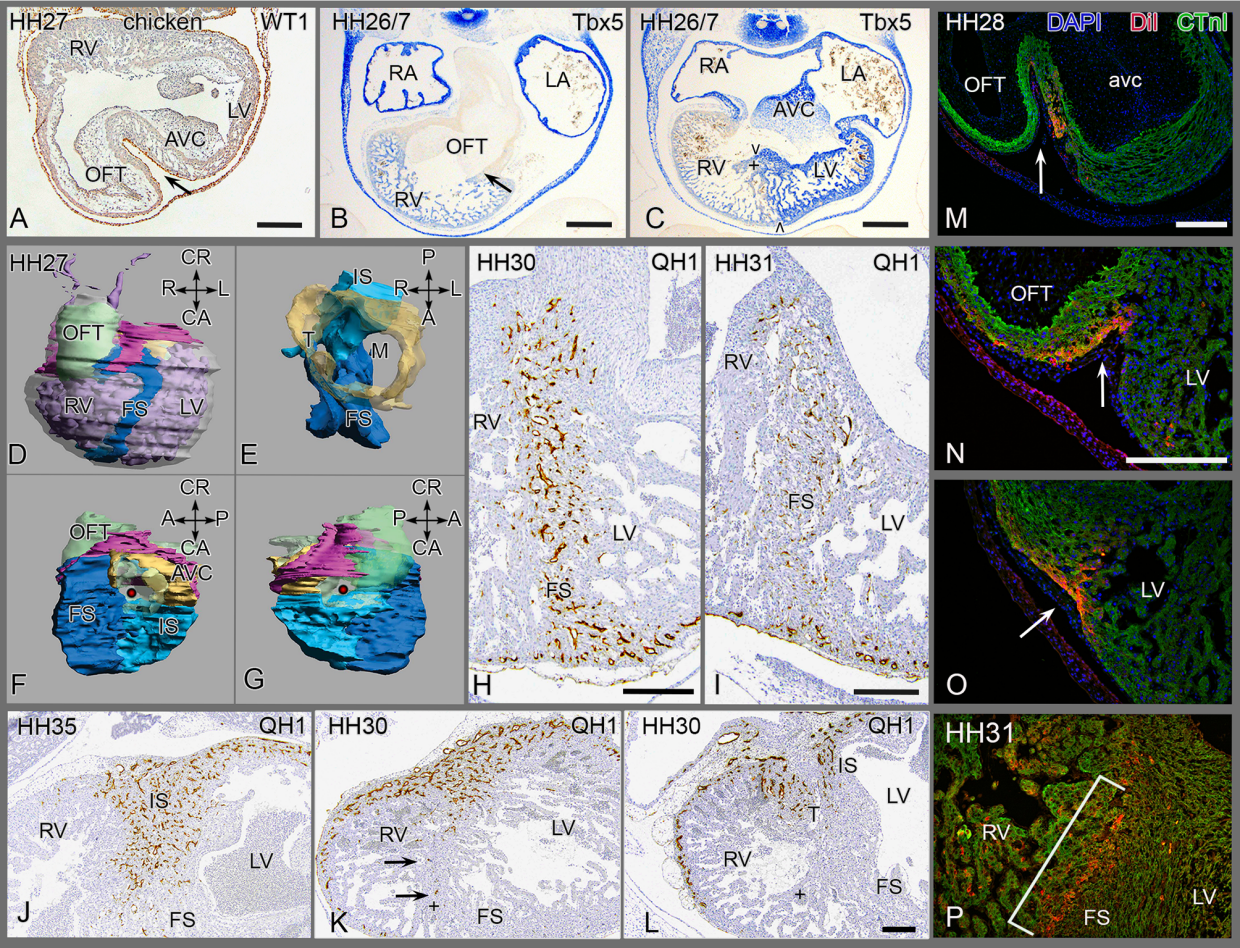
Development of chicken septum. (**A**) epicardium (→) infolding, located between OFT and AVcanal. (**B**) In situ hybridisation showing weakly positive Tbx5 of the RV and negative OFT with boundary (arrow). The atria are strongly positive. (**C**) more posterior section of the same embryo through folding septum (FS), the stronger left sided expression is evident, as is the septal band (+); boundary (> <) indicates FS. (**D-G**) 3D reconstruction with septum components and epicardial cushion. See also figure S2 for full animation and Fig. 6 for underlying sections, explaining the various components. (**H-L**) PEO quail-chicken chimeras. (**H, I**) anterior quail PEO(+liver) transplant, quail endothelial cells are exclusively present in FS and anterior free wall (**J-L**) posterior PEO (+liver) transplant with quail vascular profiles in IS (**J, K**) and right face of tricuspid orifice (**L**), but not in FS. (**K**) Several quail cells (arrows) in septal band (+), but FS does not harbor quail cells and remains negative (**K, L**). (**M-P**) DiI marking at HH17 of anterior myocardium surviving until HH28 and 31. (**M**) parts of the DiI patch (arrow) after survival to HH28 on left, (**N**) DiI on the right face and (**O**) DiI near the apex. (**P**) DiI inside the septum at HH31. Abbrev. as in Fig 2. Others: AVC atrioventricular cushions; LA/LV left atrium and ventricle; RA/RV right atrium and ventricle; + septal band.

In chicken embryos the presence of the epicardium during folding **(** **Fig. 3A** **)** is demonstrated (see also **Figure S2**), although less extensive compared to the turtle.

**Figure 4:**
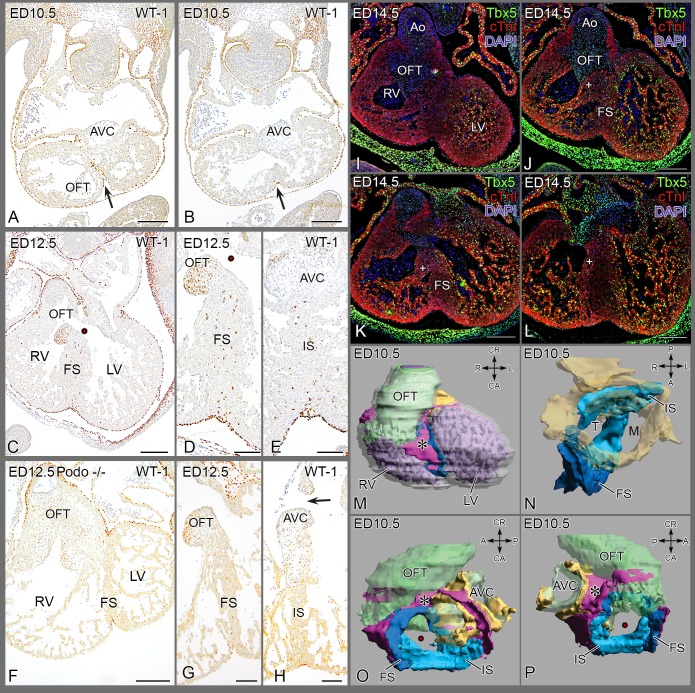
Septum formation in the mouse. (**A, B**) Almost transverse sections of the same embryo showing WT1+ epicardial cells in the folding septum (FS,→) at ED 10.5, Fig. B is more apically located. (**C, D**) epicardial cells in FS of wildtype mouse at ED 12.5 and (**E**) present in the inlet septum underneath the posterior AV cushion. Note: WT1 staining of mesenchyme in septal OFT cushion is unrelated to epicardial cells. (**F-H**) Podoplanin mutant with diminutive PEO, presents with sparse epicardium lining the pericardial cavity (**F, G**) and with an underdeveloped septum lacking EPDCs in both FS (**G**) and inlet septum (IS) (**H**). (**I-L**) Immunostained for Tbx5 in a wild type mouse ED 14.5, four levels from anterior-posterior. (**I**) Tbx5 in LV trabeculations but not in the RV close to the outflow tract; core of septum is negative. (**J-L**) More posteriorly located sections, trabeculations in RV belonging to the inlet part become positive for Tbx5. (**M-P**) Four positions of a 3D Amira reconstruction of ED 10.5. The epicardial cushion in pink (*), the folding septum in dark blue and the inlet septum in light blue. Endocardial cushions in green and the AVC myocardium in yellow. See also Fig. S3 for animated 3D. Abbrev. AVC atrioventricular cushions; FS folding septum; IS inlet septum; LV left ventricle; M mitral orifice; RV right ventricle; OFT outflow tract cushion; T tricuspid orifice, • interventricular communication, + septal band.

Development of the epicardium in mice has been extensively described [18]–[21], and here the presence of an epicardial epithelium in the folding zone **(** **Fig. 4A, B** **)** is shown (**Fig. 4M-P** **,** **Figure S3**).

**Figure 5:**
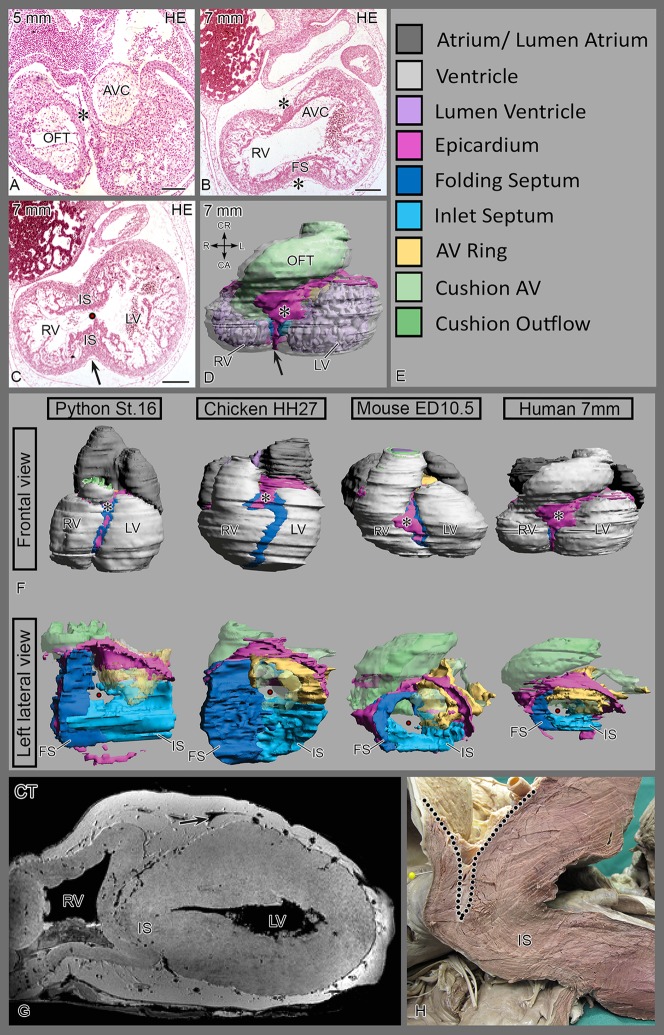
Embryonic human and adult elephant hearts. (**Fig. A, B**) Epicardial cushion (*) in inner curvature between OFT and AVC. The inlet septum becomes apparent more apically (**Fig. C**). (**D**) and **Fig S4** represent a reconstructed 7 mm embryo with folding and inlet septum formation. (**F**) 3D Comparison of development. Top row, 4 species showing relation of epicardial cushion (pink) with folding septum (dark blue). Bottom row, left lateral view, showing connection of folding septum with the inlet septum (light blue). The interventricular foramen connects left and right ventricle. In python this connection is represented by the cavum venosum. The AV myocardium is depicted in yellow and the various cushion tissues in green. (**G, H**) **adult elephant heart** (**G**) CT-image of first bifid elephant heart. (**H**) Anatomical dissection of the interventricular septum of the second heart. Left descending coronary artery (arrow) and the deep epicardial fat pad are outlined. Note the relative absence of a folding component. The dissected chordae tendineae of the tricuspid valve are visible in the lower part of Fig 5H. Abbrev. AVC atrioventricular cushions; FS folding septum; IS inlet septum; LV left ventricle; RV right ventricle; OFT outflow tract cushion.

To examine the wider implications of our model, we examined human embryos at Carnegie stages 11–15 (3.6–7 mm, **Figure S4**). The surface of the heart is covered by the epicardial epithelium while the inner curvature harbors an extensive epicardial cushion comparable to the turtle. The anterior component of the septum seems to form by folding **(** **Fig. 5A-D** **)** as in the other species (**Fig. 5F**).

In contrast, in adult elephants the folding septum is poorly developed and lacks a solid muscular core, as is evident from the deep anterior interventricular sulcus. We examined hearts with computed tomography and magnetic resonance imaging and found a deep epicardial fat pad separating the two ventricles over more than 50% of their antero-posterior extent **(see** **Fig. 5G, H** **).** Examination of the internal right septal surface revealed the septal band with tricuspid valve chordae tendineae attached, continuing as the moderator band to the free RV wall as in the human heart **(** **Fig. 1B** **).** Using these structures as landmarks, we concluded that the septum consists of an inlet component comparable to human, but the muscular walls have not fused in the folding component.

### The epicardium in the avian heart

**Figure 6:**
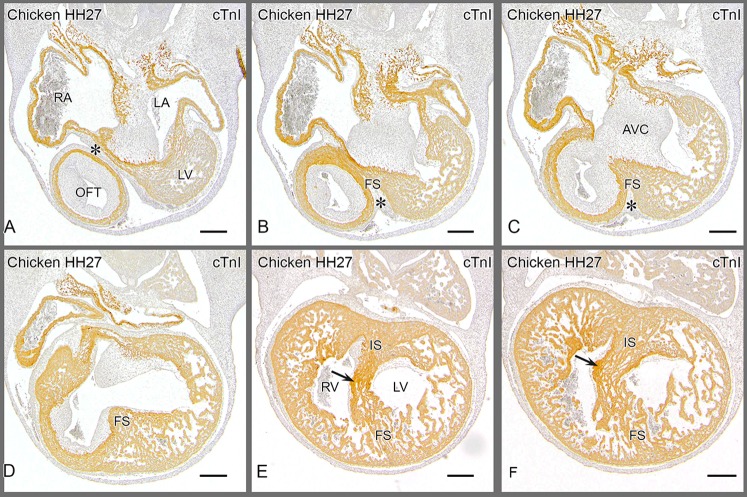
Chicken embryo HH27. Provides 6 sections of a serially sectioned chicken embryo (HH27) to demonstrate the merging of the folding and inlet components before septation is finished. From this embryo Fig 3D and Fig. S2 have been reconstructed. Similar series served as basis for the other species depicted in Fig S1, S3 and S4. **Fig 6** **.A** is most cranial, showing the epicardial cushion (*) at a level between outflow tract (OFT) and the right (RA) and left atria (LA) with the AV cushions in between. The cranial cap of the left ventricle (LV) is grazed in the section. **Fig 6** **.B** and **C** give the cranial extension of the folding septum (FS) with the epicardial cushion (*) located between the outflow tract and the fused AV cushions (AVC). **Fig 6** **.D** shows the FS bordering the interventricular foramen. It is evident that the core of the folding septum is lined on the left and right side by many trabeculations. **Fig. 6.E** The AV cushions are attached to the flanks of the inlet septum where also the tip of the septal OFT cushion is found (arrow). The inlet (IS) and folding components have fused and constitute the floor of the interventricular foramen. **Fig 6** **.E, F** The right AV junction is present in the RV immediately above the arrow and can be traced upstream in Fig F and downstream in Fig. D. Note the close relationship to the IS. The folding septum becomes less compact and the trabeculations become more conspicuous. Abbrev. AVC atrioventricular cushions; FS folding septum; IS inlet septum; LA left atrium; LV left ventricle; RA right atrium; RV right ventricle; OFT outflow tract cushion with its proximal tip indicated by arrow in Fig. F.

In the inner curvature of the early looping heart tube the bulboventricular fold is positioned between the AV canal and the OFT with, as yet, no sign of ventricular septation. Between HH22–27 by outgrowth of the left and right chambers the interventricular sulcus (**Figs. 3A, D-G**) can be appreciated as extension of the bulboventricular fold. The chambers identified in this project are depicted in a serially sectioned HH27 chicken embryo **(** **Fig. 6A-F** **)** immune-incubated for cardiac troponin I. Moving from outflow to apex, the inner curvature is present in the first section **(** **Fig. 6A** **)** containing the epicardial cushion between the OFT and AV cushions. More apically the folding septum appears **(** **Fig. 6B** **)**, as well as the interventricular communication between left and right sided chambers with the tip of the septal OFT cushion adjacent to the folding septum **(** **Fig. 6D** **)**. This is followed by the appearance of the posteriorly located inlet septum, that fuses with the anteriorly located folding septum **(** **Fig. 6E** **)**. Note the hinge point between the two septal components (arrow) and note that the septal OFT cushion ends here. Closest to the apex **(** **Fig. 6F** **)** the LV is characteristically circular in shape, and the RV crescent-like. Apically, the inlet and folding septum components become inconspicuous as the number of trabeculations increase and we refer to this component as the apical trabecular septum (Table 1). Close to the inner curvature the folding septum is trapping the epicardial cushion containing mesenchymal subepicardial cells, or more apically only an epithelial sheet of epicardium. At HH 31 this has been embedded in the heart and is no longer visible as an epithelium. The accompanying subepicardial cells or EPDCs apparently have been dispersed in the cardiac wall (see below).

To investigate the fate of the epicardium during folding we constructed quail-chicken chimeras. An isochronic quail PEO including a small piece of adjacent liver tissue to provide endothelial cells (as explained in the Materials and Methods section) was transplanted into the pericardial cavity of HH15–17 chick embryos in an anterior position, relative to the inner curvature. Using quail-specific antibodies quail EPDCs and endothelial cells were demonstrated in the folding septum, but not in the inlet septum. In later stages the quail epicardial sheet dispersed into individual cells that became distributed between the cardiomyocytes, mostly in the core of the folding septum (**Fig. 3H, I**). Both quail EPDCs (stained with the nuclear QCPN antibody) and adjacent co-transplanted endothelial cells (stained with the cytoplasmatic QH1 antibody) were encountered. They occupy the same regions in the chimera although the area occupied in sister sections by endothelial cells is slightly more restricted. Nevertheless, we have chosen to present the latter **(** **Fig. 3H-L** **)** as these are better visualized in the low magnifications needed.

In a second set of chimeras, we positioned the quail PEO dorsally to the inner curvature, leading to the presence of quail EPDCs and endothelial cells on the posterior ventricular surface, subsequently migrating into the ventricular inlet septum including the septal band, but not in the anterior folding septum. This indicates the development of the inlet septum as a separate component (**Fig. 3J-L**). An epicardial sheet, reminiscent of the anterior folding septum was not encountered, therefore, we concluded that expansion of the ventricles immediately downstream of the AV canal occurs in a ventral direction, resulting in anterior folding of the ventricular wall, but not of the posterior wall, probably because of the physical constraints imposed by the dorsal body wall. In HH30 the right (tricuspid) opening in the AV canal is visible and separated from the mitral orifice. The right AV orifice is flanked on the left-side by the inlet septum and on the right-side by the RV wall. After chimerization both sides contain quail cells that have migrated into these parts of the cardiac wall **(** **Fig. 3L** **)**.

To study the folding mechanism fluorescent DiI was applied to the surface of the myocardium [22] ventrally to the inner curvature in HH 15–17 chicken embryos before epicardial covering, and embryos were sacrificed between HH22–33 **(** **Fig. 3M-P** **)**. Fluorescent patches positioned exactly in the future fold separated during further development into a left-sided fragment, incorporated in the left side of the folding septum (**Fig. 3M**) and a right-sided fragment (**Fig. 3N**) extending to the surface towards the apex (**Fig. 3O**). This indicates a longitudinally directed morphogenetic expansion of the right ventricular wall [23] compared to a transverse expansion of the left wall [22]. At stage 31 the DiI labelled myocardial cells were completely embedded in the folding septum as visualized by a narrow fluorescent strip located close to the right ventricular face of the septum (**Fig. 3P**), indicative of a more massive contribution of the left ventricular wall to the septum.

### Disturbance of the epicardium

Development of the epicardium in mice has been analyzed in recent years [18]–[21]. As the folding and epicardial incorporation is very similar to avian development it will not be treated separately. Deficient septation, however, is known in several animal models including PEO ablation in chicken embryos [25] and mouse mutants and we have chosen to analyze the podoplanin mutant mouse. Podoplanin is expressed in the lining of the body cavities including the epicardium and pericardium. The podoplanin mutant mouse has been morphometrically analysed [7] and presents with an underdeveloped PEO (40% compared to wildtype) and abnormal epicardial covering with hardly epithelial to mesenchymal transition resulting in only a few EPDCs. Analysis of this PEO-deficient mouse shows multiple malformations including an atrioventricular septum defect and a thin myocardium. At embryonic day (ED) 12.5 the folding septum is very thin, and the inlet septum spongy. The diminutive septum is nearly devoid of EPDCs (**Fig. 4C-H**), suggestive of an instructive role for these cells in completion of the septum.

The 3-D reconstruction of a wild type mouse embryo is provided in **Fig. 4M-P**.

### Septum components in reptilian hearts

The horizontal septum (syn. muscular ridge, “Muskelleiste”) is found in similar developmental stages, in the same location, separating ventricular cavities, and harboring an epithelial epicardial sheet much the same as the folding septum in mammals and birds. The mentioned names have been used interchangeably but we prefer to address this structure as ‘folding septum’ **(** **Table 1** **)**.

The presence and extent of the vertical septum (homologous to inlet septum) differs among turtles and squamates, being virtually absent in turtles (**Fig. 2I**), but being more prominent in varanids and pythonidae (**Figs. 2E-G, K**). Myocardial apical trabeculations (**Fig. 2G**) traversed the cavum dorsale connecting the anterior and posterior myocardial walls, partly separating the cavum dorsale into the cavum venosum and arteriosum, but at a different position compared to the folding septum.

### Tbx5 expression patterns

The T-box transcription factor Tbx5 has been reported to be expressed in the heart from left to right in a gradient that declines towards the right side [14]. Tbx5 expression in the early embryonic copperhead rat snake, *Coelognathus* (**Fig. 2H**) was evident in the cavum dorsale and absent in the OFT. In the embryonic turtle (**Fig. 2I**) as well as in the python (**Fig. 2J, K**) there was a boundary at the folding septum and ventricle. Tbx5 thus exhibited a distinct decline in expression only at the cavum dorsale/OFT boundary (**Fig. 2J**). Its expression was uniform over the three cava in the direction of the apex (**Fig. 2K**). It is obvious that in the turtle the tip of the trabeculations show a stronger Tbx5 expression than the adjacent tissues (**Fig. 2I**).

In the chicken the Tbx5 mRNA gradient identifies the RV(weak expression) and the Tbx5-negative OFT (**Fig. 3B**). Additionally, a second Tbx5 gradient is found at the folding septum showing strong expression in the LV but weak in the RV (**Fig. 3C**), whereas the expression is present on both sides of the inlet septum including the septal band. Thus, the two components of the ventricular septum in chicken, namely folding and inlet septa (**Fig. 3D-G**) are differentially identified by Tbx5 gradients.

In the mouse similar to the chicken, two Tbx5 gradients identify the inlet and folding septum as exemplified by the protein pattern **(** **Fig. 4I-L**). Protein expression is strongest in the trabeculations of the LV (**Fig. 4I, J**), weaker in the RV inlet including septal band, **(** **Fig. 4** **J, K)** and weakest to negative in the RV OFT **(** **Fig 4I** **)**.

## Discussion

The evolution from the single fish ventricle to the two ventricles of mammals and archosaurs is the result of ventricular septation and has been studied and discussed for more than a century. It is tempting to apply to intermediate stages the partially septated ventricles encountered in turtles, lizards and snakes. Indeed, all amniotes exhibit a horizontal or folding septum and here we provided a mechanism for its formation involving the epicardium in fusion of two opposing myocardial walls. In the completely septated ventricle, the folding septum forms the anterior part of the septum. The epicardium originating from the PEO [24]–[26], important for multiple aspects of heart development, is already present in lampreys [27]. Reducing the size of the PEO is exemplified by the podoplanin mouse mutant [7] and inhibits its outgrowth in chicken [6]. Both lead to diminished or retarded covering of the myocardium and lack of epithelial-mesenchymal transition producing a decreased number of EPDCs (this paper). Many genes are expressed in the epicardium lining the myocardium of the heart. They can be divided in ‘epithelial genes’ including integrins and b-catenin, but also others like RALDH2, and several transcription factors [28] including Tbx18, Tbx5, Tcf21, NFATc1, and WT1. A subset of these genes is important for epithelial to mesenchymal transition resulting in the formation of EPDCs. The latter can differentiate into smooth muscle cells of the coronary vessels and into perivascular and interstitial fibroblasts. It is evident that mechanical or genetical interference with the epicardium or EPDCs not only disturbs coronary vascularisation but can strongly influence cardiomyocyte differentiation and ventricular septation [6], [7]

Different septal components have been identified in the completely septated hearts but the origin and subdivision of the primary interventricular septum has been the subject of continued debate [8]–[10], [29], with varying implications for the explanation of the position of central muscular ventricular septal defects, the origin of the left and right bundle branch and the connection of the tricuspid valve tendinous cords related to either septal components. It was generally agreed that the septal band belonged to an anterior component of the septum, the primary septum, but we describe it as a derivative of the posterior inlet septum. In normal hearts but also in patients with septal defects [3] the position of the AV valve leaflets and the connection of the tension apparatus to the septal band and the inlet septum is consistent with our evolutionary and developmental concept. We could identify the border between inlet and folding component according to the combination of the following criteria: (i) the AV cushions are connected to the inlet component including the septal band; (ii) the proximal tip of the septal OFT cushion is incorporated in the zone where the inlet septum merges with the folding septum; (iii) quail cells derived from anterior PEO chimeras do not crossover from folding septum to inlet septum (including septal band) and, likewise, there is no crossover in posterior chimeras from inlet septum (including the septal band) to folding septum; (iv) Tbx5 expression is strong in the complete inlet septum (both left and right side) including the septal band, whereas the folding septum only shows left-sided positivity consistent with this part belonging to the primitive embryonic ventricle. These combined characteristics support the concept that the early embryonic ventricle in birds and mammals (homologous to the cavum dorsale in reptiles) gives rise to both the LV and inlet of the RV, also implicating that the inlet septum originates in its entirety from the wall of the primitive ventricle (the cavum dorsale) and becomes partitioned over the LV and the RV inlet. The origin of the trabeculated apical portion of the interventricular septum has not been approached by our experiments but it can be traced back in the primary heart tube of chicken [30]. In the RV the antero-posterior boundary is determined by the septal band, which is lacking in the LV, leaving the LV boundary less well determined.

Our new model for partitioning of the ventricles comprising more than one component (note: the membranous septum related to the AV cushions, and the muscular OFT septum related to the neural crest, have not been specifically studied here) has consequences for the development of AV and muscular ventricular septal defects at the border of septal components.

Interestingly, the curious ‘bifid’ heart of elephants, seacows [31] and some other marine mammals may be interpreted as the retention of an embryonic feature resulting from non-progressed fusion of the ventricular walls of the folding septum, rather than regression from a well-developed septum. Obviously, the small coney or rock hyrax (*Procavia capensis*), a close extant relative to the elephant and the seacows, does not present a bifid heart (unpublished).

The development of both the folding and inlet components with differently incorporated EPDCs is severely hampered in mutant mice deficient for epicardially expressed genes.

This may lead to an abnormal septum [7], [32], [33] and to multiple muscular ventricular septal defects, reported in human congenital heart disease [3] as a result of deficient myocardial compaction [19].

### Strengths and weaknesses

An important novelty of this study is found in distinguishing several septal components each with their own characteristic developmental history and mechanistic program. The exact boundaries in the fully septated heart, however, remain difficult to define. It is a limitation of the study that no specific tissue markers are available, implying that we rely on the combination of various approaches, each with their own strength and weakness. We are of the opinion that combining the different techniques and referring to data in which their combination supports the conclusions, reduces substantially erroneous interpretations and allows us to add substantial new evidence to earlier studies that postulated the existence of more than one ventricular septal component.

In conclusion, we have added in an evo-devo context a hitherto overlooked, but important role for the epicardium in ventricular septation and have clarified complex homologies of the ventricular septum in amniotes aiding to understand clinical disorders of the heart in humans.

## Materials and Methods

### Animal material

Animal material was obtained as follows. Mouse embryos were harvested between embryonic day 12.5–15.5 from strains housed in the fully licensed Animal Facility of the Leiden University Medical Center. Pregnant dams were killed by cervical dislocation. The podoplanin mouse material was obtained in a collaboration and provided by P. Uhrin (Vienna). Animal care and all experimental procedures were approved by the Animal Experimental Committee of the Medical University of Vienna, and by the Austrian Ministry of Science (License No. 1321/115, and 66.009/0103-C/Gl/2007). Fertilized chicken eggs were obtained from a commercial breeder. Chicken and reptile eggs are not considered experimental animals under the Dutch law. The oldest chicken stages harvested after manipulation are Hamburger Hamilton stage 34. All reptile embryos were obtained legally from general-purpose captive-bred populations and were harvested and fixed in conformity with local and international regulations. Embryos were euthanized instantly by fixation to cause minimum suffering. Their use was approved according to the Regulations of the Animal Experimental Committee of the LUMC and Leiden University based legally on the national “Wet op de Dierproeven” (Article 9). This regulation serves as the implementation of Guidelines on the protection of experimental animals by the Council of Europe, Directive 86/609/EEC. Stages used were before 35% of embryonic development prior to hatching, which is in conformity with the local, national and European Union regulations. The two available elephant hearts were obtained after euthanasia of the animals for unrelated metabolic or arthritic disease and made available by the dept. Veterinary Pathology (M. Kik, University of Utrecht) with a licence for the study of protected animal material. The anonymized human embryos belong to the collection of the department of Anatomy (Vienna) and have been photographed over 3 decades ago for peer reviewed publications on development.

### Quail-chicken chimeras

To study EPDCs and endothelial (precursor) cell migration in more detail, chimeras were generated using quail (*Coturnix coturnix*) embryos as donors and White Leghorn chicken embryos as hosts. Embryos were staged [34]. Quail-chicken chimeras were made as described [35]. In brief, the PEO of a HH15–18 quail embryo was isolated together with a tiny piece of liver tissue to provide endothelial precursor cells to the PEO transplant and transplanted into the pericardial cavity of a HH15–18 chicken host embryo through the naturally occurring hiatus in the body wall that exists until HH18. The transplant was positioned along the developing heart tube, either dorsal or ventral in the inner curvature. Chimeras (n = 31) were harvested between stage HH19 and HH32.

### Histological Procedures

Normal and chimeric embryos were fixed by overnight immersion in 4% paraformaldehyde (PFA) in 0.1 M phosphate buffer (pH 7.4) at 4°C. Serial sections were immunostained. Tissue processing and immunohistochemistry were described [7].

### DiI labelling in chicken

White Leghorn chicken (*Gallus gallus domesticus*) eggs (n = 36 analysed) were incubated at 38°C on stationary shelves until stage 18–19 [36]. The outer face of the looping heart tube was tattooed with a 1.25 mg/ml red fluorescent mix of 1,1′-dioctade dioctadecyl-3,3,3′-tetramethylindocarbocyanine perchlorate (DiI, Invitrogen, D-282) and 5-carboxytetramethylrhodamine, succinimidyl ester (TAMRA SE, Invitrogen, C2211) as described [36]. Localized microinjection, using an ultrathin glass needle (inner diameter 0.01 mm) and a Narishige IM-300 microinjector, of a minimal fluorophore volume at the anterior surface near the inner curvature resulted in labelling mostly myocardial but also epicardial cells. After microinjection, the eggs were re-incubated to allow further development to stages 24–35.

### Mouse podoplanin

Mouse embryos were generated and obtained in collaboration with P.Uhrin (Vienna). After staging of pregnancy the dams were killed and embryos harvested and fixed. Serial sections were immunostained for the myocardial marker MLC2-a (1/6000, kindly provided by S.W.Kubalak, Charleston, SC, USA) and WT-1 (1/1000, Santa Cruz Biotechnology, CA, USA) as previously described [7].

### Mouse Tbx5 immunochemistry

For immunofluorescent detection of Tbx5, 6 µm serial sections of wild type embryos were deparaffinized and rehydrated. After boiling in Antigen Unmasking Solution (H-3300, Vector Laboratories) in a pressure cooker for antigen retrieval, sections were blocked in 1% bovine serum albumin (Sigma) in 0,1% Tween-PBS for 30 minutes and incubated O/N at 4°C. Next, the sections were incubated with Tbx5 antibody (a kind gift from C.J.Hatcher, Ithaca, NY) and subsequently with horseradish peroxidase-conjugated secondary antibody, followed by Tyramide Signal Amplification (Perkin Elmer Life Science; #NEL700A). Signal was visualized using Alexa 488-conjugated streptavidin (Invitrogen, S-11223). Subsequently, (sister) sections were incubated with antibodies directed against cardiac Troponin I (cTNI, HyTest Ltd, CN-4T21_2) followed by Alexa 555-conjugated secondary antibodies (Invitrogen, A-21432) to visualize the myocardium. Sections were mounted using ProLong Gold antifade reagent (Invitrogen, CN-P36930) with DAPI.

### Whole mount chicken and reptile Tbx5 mRNA

This has been performed as described [37], [38].

### CT scanning

This was performed on two adult Asian elephant (*Elephas maximus*) hearts using a 64 slice CT scanner (Toshiba Aquilion, Toshiba Medical Systems, Otawara, Japan). Helical acquisition settings were tube voltage 100 kV, tube charge 500 mAs, and pitch 0.83. Reconstruction with slice thickness 0.5 mm, FOV 500 mm, 512×512 matrix and a soft tissue reconstruction filter (FC03). 3D Visualisation by using volume rendering (Osirix v5.6). We examined the hearts with computed tomography and magnetic resonance imaging and found a deep epicardial fat pad separating the two ventricles over more than 50% of their antero-posterior extent.

### SEM protocol

Hearts of embryos of the pythonid snake *Liasis mackloti* were fixed in 70% ethanol. The vessels at the venous pole of the heart were removed and hearts dissected prior to critical point drying with a BAL-TEC Critical Point Drier 030. The samples were sputter coated with a gold layer by using a Polaron E5100 and were analysed with a JEOL scanning electron microscope.

### Amira protocol

Three-dimensional reconstructions of snake, chicken, mouse and human hearts were made. Photographs of serial sections stained for cTnI (snake, chicken mouse) or HE (human) were taken with an Olympus Provis AC70 microscope fitted with Olympus UPlan Apo-objectives, using an Olympus XC50 camera. Every 5th section of 5 µm was used. The sections in between were stained with various other antibodies such as WT1. Pictures were optimized for Amira version 5.4.2 with Adobe Photoshop CS6 Extended. See **Figure 6** as example using the chicken. Sections were both automatically and manually aligned and labels were added to the different structures, based on morphology and stains. Surface views were executed to PDF formats by using Adobe Acrobat 9.5 Extended. The animation of Amira files with help of Adobe pdf (version 11 or higher is needed for full functionality) has been described [39].

## Supporting Information

Text S1 Figs. S1–S4 present the fully animated reconstructions of python, chicken, mouse and human hearts, respectively. Comparison between the four species is provided in main text figure 5F from a frontal and left lateral view. A short manual how to use the animated Adobe pdfs (version 11 or higher is needed). The color legend provided (Fig 5E) is useful to recognize the various structures. Moreover, several structures in the animated reconstructions have been annotated for your convenience.
(DOCX)

Figure S1 **Animated pdf of an embryonic python heart (Liasis mackloti).** The inlet septum is indicated in light blue and the folding septum in dark blue. In this stage, the epicardium (pink) is mainly associated with the AV-ring (yellow) and the folding septum. Endocardial cushion sets (OFT and AV) are represented in shades of green.
(PDF)

Figure S2 **Chicken embryo HH27.** Animated pdf of the same chicken embryo of Fig. S2. The inlet septum is indicated in light blue and the folding septum in dark blue. In this stage, the epicardium (pink) is mainly associated with the AV-ring (yellow) and the folding septum. Endocardial cushion sets (OFT and AV) are represented in shades of green.
(PDF)

Figure S3 **Animated pdf of a mouse embryo.** The inlet septum is indicated in light blue and the folding septum in dark blue. In this stage, the epicardium (pink) is mainly associated with the AV-ring (yellow) and the folding septum. Endocardial cushion sets (OFT and AV) are represented in shades of green.
(PDF)

Figure S4 **Animated pdf of a human embryo.** The inlet septum is indicated in light blue and the folding septum in dark blue. In this stage, the epicardium (pink) is mainly associated with the AV-ring (yellow) and the folding septum. Endocardial cushion sets (OFT and AV) are represented in shades of green.
(PDF)
