# Supplementary material for: Evolution and Development of Ventricular Septation in the Amniote Heart
Source: PLoS One. 2014 Sep 5;9(9):e106569. doi: 10.1371/journal.pone.0106569 (PMC4156344; doi:10.1371/journal.pone.0106569)
Supplement: Text S1 — Figs. S1–S4 present the fully animated reconstructions of python, chicken, mouse and human hearts, respectively. Comparison between the four species is provided in main text figure 5F from a frontal and left lateral view. A short manual how to use the animated Adobe pdfs (version 11 or higher is needed). The color legend provided (Fig 5E) is useful to recognize the various structures. Moreover, several structures in the animated reconstructions have been annotated for your convenience. (DOCX) [file pone.0106569.s001.docx]

1.Double click on the file name to activate the picture (may take a few seconds).

2.Activate the grey buttons on the left to explore preset annotated views. After activating all of them they move smoothly from one to another after re-clicking. You can rotate the object keeping the left mouse button pressed. Using the mouse wheel the object can be zoomed in/out.

3.Alternatively, click on the figure and a toolbar will appear on the top for full manipulation of the objects and the structures within. Several options are available in the toolbar. A versatile option is the following.

4.Click on the model tree close to the center of the toolbar, two windows appear on the left.

5.In the top window activate the pull down menu by clicking on the + box next to <Root>, and subsequently next to NO, and several boxes appear by name (atrium, ventricle etc.). By clicking on the boxed V adjacent to the name this structure (dis)appears. By right clicking on the name itself a submenu appears allowing other representations e.g. transparency.

6.In the lower left window the preferred views are stored for your convenience.

7.Manipulations 4 and 5 can be used independently and together, adding/hiding structures to preferred views.
