# Supplementary figures and images for: Evolution and Development of Ventricular Septation in the Amniote Heart
Source: PLoS One. 2014 Sep 5;9(9):e106569. doi: 10.1371/journal.pone.0106569 (PMC4156344; doi:10.1371/journal.pone.0106569)

# Python St 16

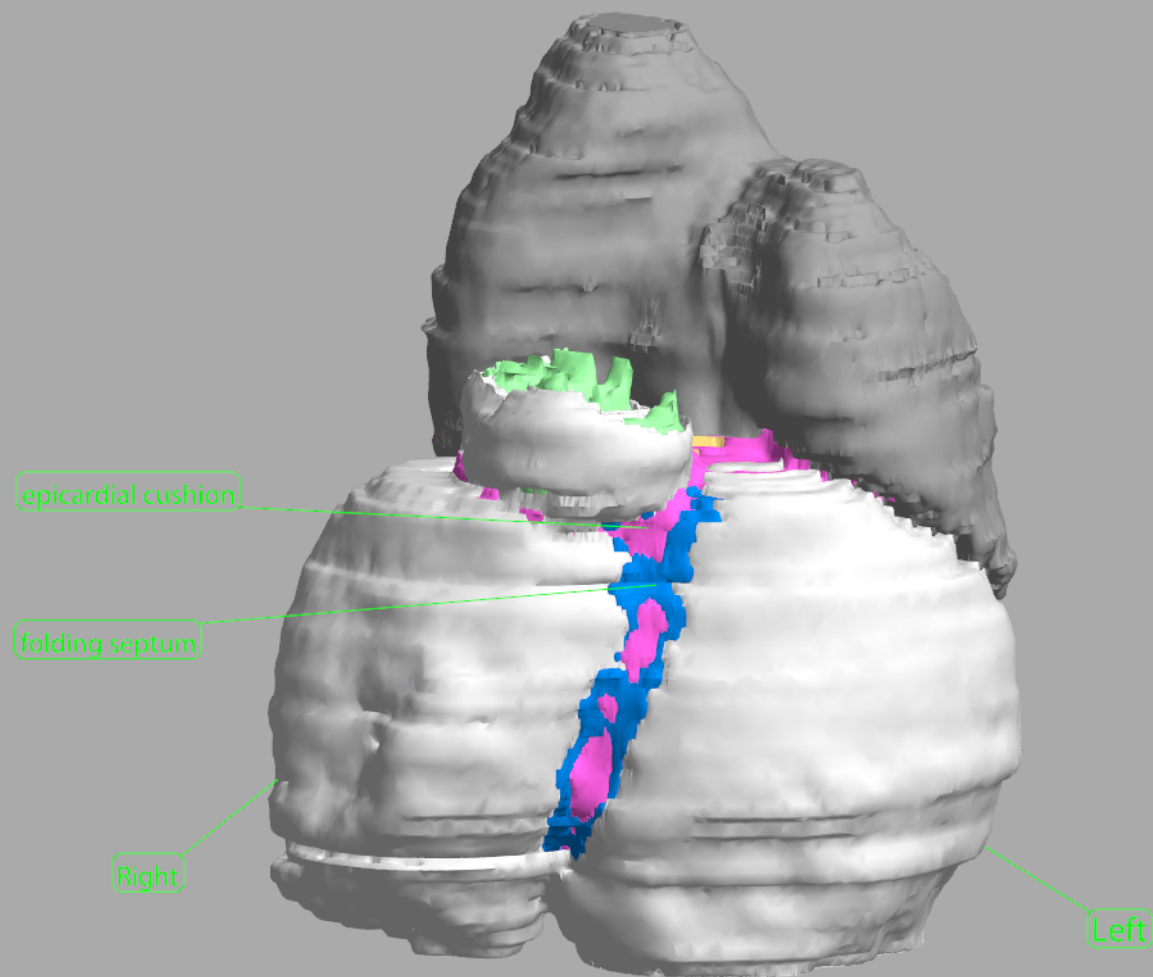

Supplement: Figure S1 — Animated pdf of an embryonic python heart (Liasis mackloti). The inlet septum is indicated in light blue and the folding septum in dark blue. In this stage, the epicardium (pink) is mainly associated with the AV-ring (yellow) and the folding septum. Endocardial cushion sets (OFT and AV) are represented in shades of green. (PDF) [file pone.0106569.s002.pdf]

# Chicken HH27

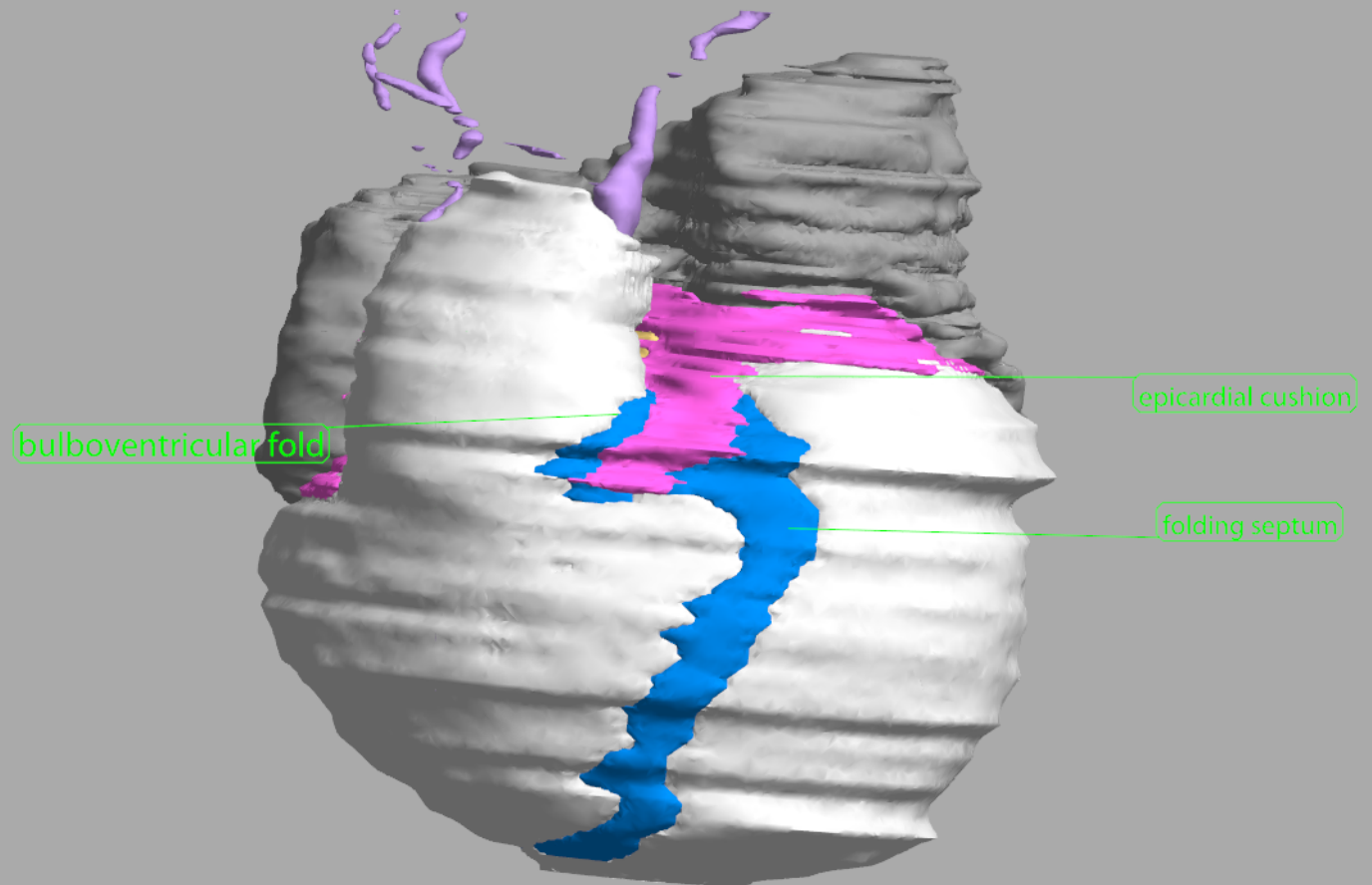

Supplement: Figure S2 — Chicken embryo HH27. Animated pdf of the same chicken embryo of Fig. S2. The inlet septum is indicated in light blue and the folding septum in dark blue. In this stage, the epicardium (pink) is mainly associated with the AV-ring (yellow) and the folding septum. Endocardial cushion sets (OFT and AV) are represented in shades of green. (PDF) [file pone.0106569.s003.pdf]

# Mouse E10.5

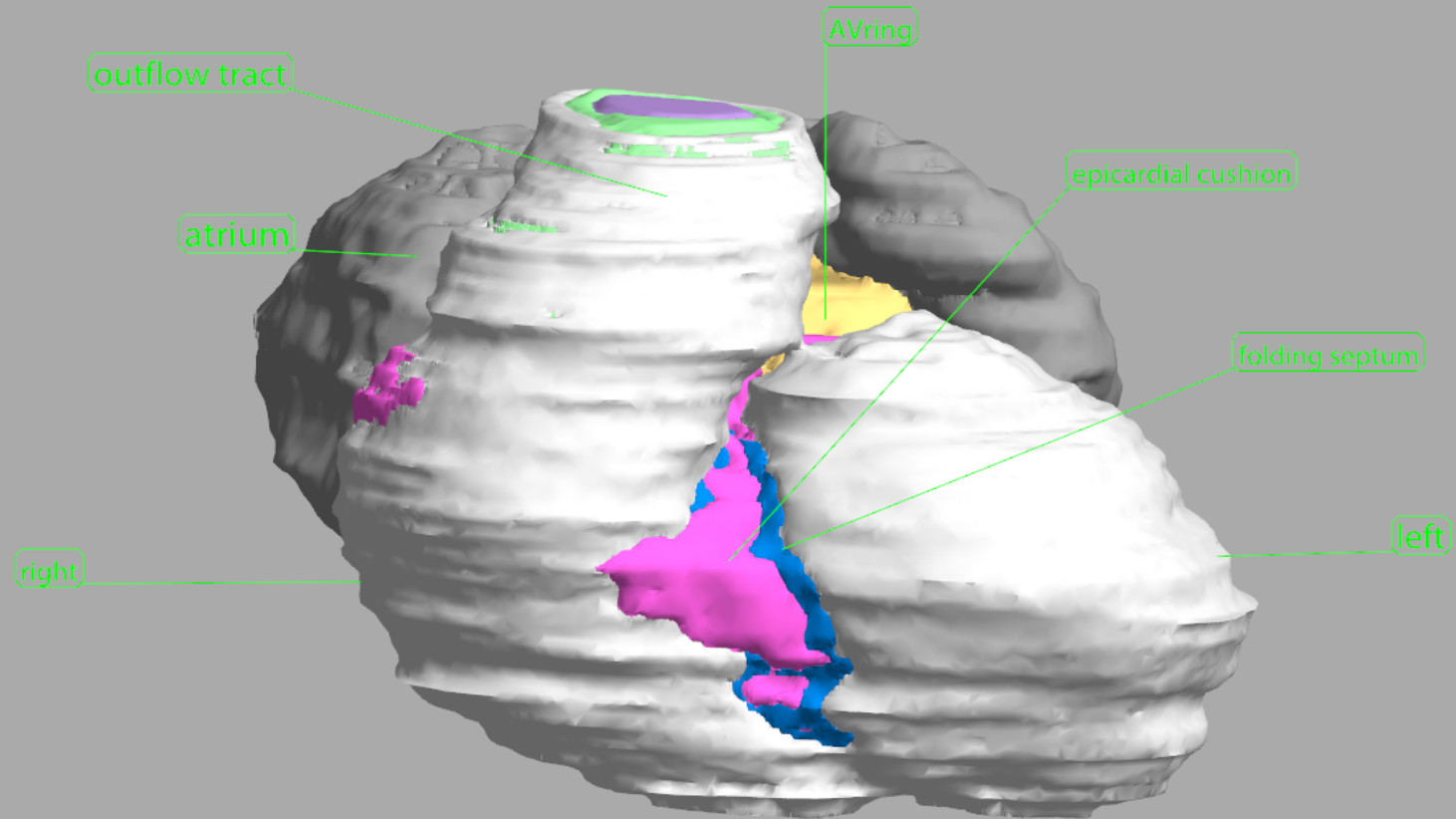

Supplement: Figure S3 — Animated pdf of a mouse embryo. The inlet septum is indicated in light blue and the folding septum in dark blue. In this stage, the epicardium (pink) is mainly associated with the AV-ring (yellow) and the folding septum. Endocardial cushion sets (OFT and AV) are represented in shades of green. (PDF) [file pone.0106569.s004.pdf]

# Human 7mm

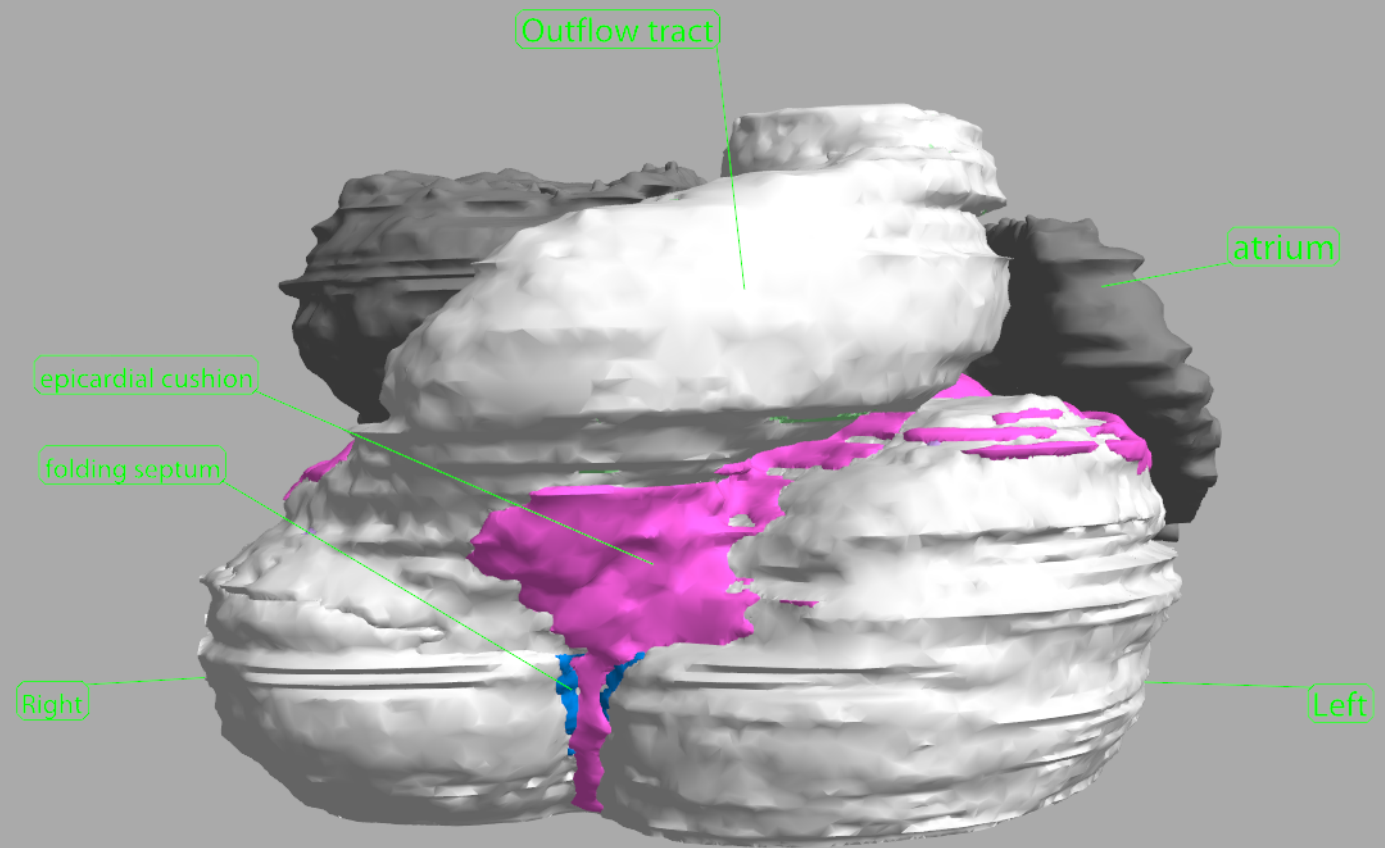

Supplement: Figure S4 — Animated pdf of a human embryo. The inlet septum is indicated in light blue and the folding septum in dark blue. In this stage, the epicardium (pink) is mainly associated with the AV-ring (yellow) and the folding septum. Endocardial cushion sets (OFT and AV) are represented in shades of green. (PDF) [file pone.0106569.s005.pdf]
